# Development of a qPCR Strategy to Select Bean Genes Involved in Plant Defense Response and Regulated by the *Trichoderma velutinum* – *Rhizoctonia solani* Interaction

**DOI:** 10.3389/fpls.2016.01109

**Published:** 2016-08-04

**Authors:** Sara Mayo, Eleonora Cominelli, Francesca Sparvoli, Oscar González-López, Alvaro Rodríguez-González, Santiago Gutiérrez, Pedro A. Casquero

**Affiliations:** ^1^Research Group of Engineering and Sustainable Agriculture, Department of Agrarian Engineering and Sciences, Natural Resources Institute, University of LeónLeón, Spain; ^2^Institute of Agricultural Biology and Biotechnology, Consiglio Nazionale delle RicercheMilan, Italy; ^3^Area of Microbiology, University School of Agricultural Engineers, University of LeónPonferrada, Spain

**Keywords:** biotic stress, systemic acquired resistance, induced systemic resistance, hypersensitive response, defense genes, biocontrol agent, *Phaseolus vulgaris*

## Abstract

Bean production is affected by a wide diversity of fungal pathogens, among them *Rhizoctonia solani* is one of the most important. A strategy to control bean infectious diseases, mainly those caused by fungi, is based on the use of biocontrol agents (BCAs) that can reduce the negative effects of plant pathogens and also can promote positive responses in the plant. *Trichoderma* is a fungal genus that is able to induce the expression of genes involved in plant defense response and also to promote plant growth, root development and nutrient uptake. In this article, a strategy that combines *in silico* analysis and real time PCR to detect additional bean defense-related genes, regulated by the presence of *Trichoderma velutinum* and/or *R. solani* has been applied. Based in this strategy, from the 48 bean genes initially analyzed, 14 were selected, and only *WRKY33, CH5b* and *hGS* showed an up-regulatory response in the presence of *T. velutinum*. The other genes were or not affected (*OSM34*) or down-regulated by the presence of this fungus. *R. solani* infection resulted in a down-regulation of most of the genes analyzed, except *PR1, OSM34* and *CNGC2* that were not affected, and the presence of both, *T. velutinum* and *R. solani*, up-regulates *hGS* and down-regulates all the other genes analyzed, except *CH5b* which was not significantly affected. As conclusion, the strategy described in the present work has been shown to be effective to detect genes involved in plant defense, which respond to the presence of a BCA or to a pathogen and also to the presence of both. The selected genes show significant homology with previously described plant defense genes and they are expressed in bean leaves of plants treated with *T. velutinum* and/or infected with *R. solani*.

## Introduction

The common bean (*Phaseolus vulgaris* L.) is the most important food legume crop worldwide. Bean production is often affected by biotic and abiotic factors (Guerrero-González et al., 2011) by microorganisms, humidity, temperature… that are detected as signals for the activation of plant response mechanisms. This crop is affected by a wide diversity of fungal pathogens (*Sclerotinia* spp., *Fusarium* spp., *Phytium* spp., *Botrytis* spp.,...) among them *Rhizoctonia solani* JG Kühn [Teleomorph: *Thanatephorus cucumeris* (AB Frank) Donk] has a remarkable importance as responsible of important economic losses in this crop (Valenciano et al., 2006). *R. solani* is a necrotrophic pathogen responsible for the root and hypocotyl diseases. Plant infection occurs through wounds or by the direct action of the fungal mycelium, which tears the cuticle and penetrates the epidermis (Guerrero-González et al., 2011).

As a strategy to control bean infectious diseases, mainly those caused by fungi, the use of biocontrol agents (BCA) can reduce the negative effects of plant pathogens and they also can promote positive responses in the plant (Shoresh et al., 2010). The genera *Trichoderma, Gliocladium, Rhizobium, Pseudomonas*, are beneficial organisms that have shown good efficiency as BCAs against pathogenic microorganisms. *Trichoderma* (Teleomorph: *Hypocrea*) is a fungal genus that is found in the soil, and it is a secondary fast growing opportunistic invasive. In addition, *Trichoderma* biocontrol strains are able to induce the expression of genes involved in defense response and also to promote plant growth, root development, and nutrient uptake (Hermosa et al., 2012).

The relationships established between plant and micro-organisms are very diverse. When a plant is exposed to a pathogenic microorganism, the production of molecules associated to salicylic acid is increased, being this a systemic acquired resistance (SAR) response. The response of plants against non-pathogenic microorganisms is different, resulting in activation of signaling cascades that are dependent on jasmonic acid and ethylene, such as hydroperoxide lyase, peroxidase, and phenylalanine ammonia lyase, all of which belong to an induced systemic resistance (ISR) response (Druzhinina et al., 2011). Other responses result in a rapid cell death in infected tissues, then plants activate the hypersensitive response that involves the accumulation of salicylic acid, reactive oxygen species and an increased the influx of Ca^2+^ (Guerrero-González et al., 2011).

In the tripartite interaction of bean plants with the pathogen *R. solani* and a biocontrol *Trichoderma* species, several changes are produced in the plant, such as the increase in phenolic acid and lignin, accumulation of phytoalexins (Guerrero-González et al., 2011), and down- or up-regulation of defense-related genes expression (Mayo et al., 2015). Different categories of defense-related genes whose expression is modulated by biotic stresses have been described in bean plant interacting with pathogen and non-pathogenic microorganisms (Mayo et al., 2015).

Our hypothesis is that the combination of real time PCR with “*in silico*” analysis is a valid strategy to identify bean defense-related genes regulated by BCAs and/or plant pathogens. The aim is develop a systematic strategy to detect bean defense-related genes regulated by the presence of *Trichoderma velutinum* and/or *R. solani.* Finally, the procedure has been validated by the analysis of expression of the selected genes in the presence or absence of these two fungi.

## Materials and Methods

### *Trichoderma* and *Rhizoctonia solani* Isolates and Culture Collections

**FIGURE 1:**
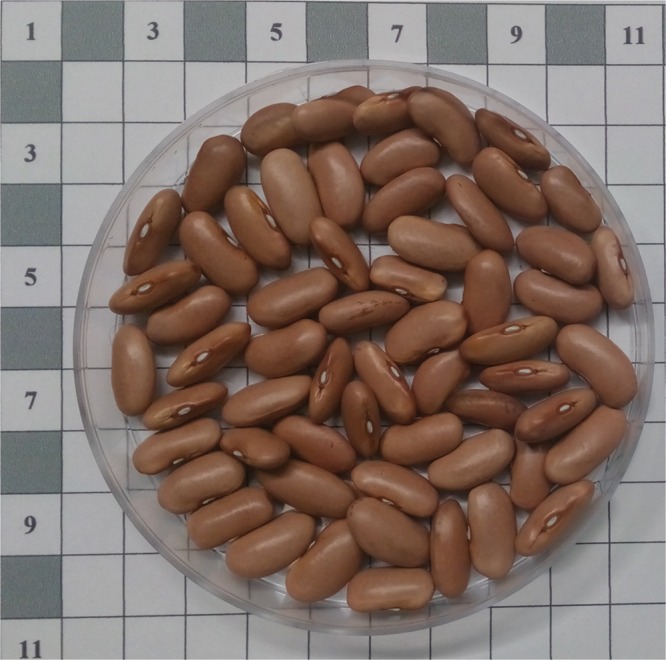
**Bean seeds of “Canela” variety of the Protected Geographical Indication “Alubia La Bañeza – León” (Spain)**.

*Trichoderma velutinum* T028, was collected from the bean traditional production area (Protected Geographical Indication, PGI), called “Alubia La Bañeza - León” (EC Reg. n.256/2010 published on March 26th, 2010, OJEU L880/17), from a High Quality variety of beans (**Figure 1**) without any genetic manipulation. It was isolated from soil plot bean in the Astorga region (León, Spain). This isolate gave percentages of inhibition greater that 60% in membrane assays and 40% in direct confrontation assays with *R. solani*, and that was able to sporulate on potato-dextrose-agar (PDA) medium.

*Rhizoctonia solani* R43 was isolated from bean plants of the same PGI and selected based on its high virulence. The isolated strains were stored in the collection “Pathogens and Antagonists of the Laboratory Diagnosis of Pests and Diseases” (PALDPD, University of León, León, Spain).

Isolates were inoculated on PDA (Becton Dickinson, Germany) medium and grown at 25°C in the dark for 1 week. After this incubation time *T. velutinum* T028 was exposed to light in order to induce the spore’s formation.

### Plant Materials and Growth Conditions

Sixty bean seeds (Canela landrace, PGI “Alubia de la Bañeza –León,” **Figure 1**) per treatment were germinated and cultured in presence or absence of the fungi in four conditions according to the procedure previously described by Mayo et al. (2015): (i) *T. velutinum* (T028) isolate plus *R. solani* (R43) (RT028); (ii) *T. velutinum* isolate (T028) without pathogen (C = control) (CT028); (iii) control (without *T. velutinum*) with *R. solani* (RC) and (iv) control without fungi (CC). The culture was carried out in climatic chamber and growth conditions were performed as previously described (Mayo et al., 2015). Six bean leaves from 45 day-old plants of each treatment were randomly collected and stored at -80°C until use.

### RNA Extraction and Purification

The procedures for RNA extraction were performed as described previously (Reid et al., 2006). Bean leaves were lyophilized and were ground to a fine powder in liquid nitrogen using a mortar and pestle. The powder was mixed with 20 ml of extraction buffer/g of sample (extraction buffer: 0.1% SDS, 100 mM LiCl, 10 mM EDTA, 100 mM Tris-HCl, pH9) pre-warmed at 65°C, and 20 ml/g of phenol-chloroform-isoamyl alcohol 25:24:1 (Sigma–Aldrich, St. Louis, MO, USA). Then, the mixtures, in eppendorf tubes, were centrifuged at 13,000 rpm for 10 min at 4°C. The aqueous layer was transferred to a new tube. This step was repeated twice. Nucleic acids were precipitated with 1 volume of LiCl 4 M, mixed and kept overnight at 4°C. Tubes were then centrifuged at 13,000 rpm for 30 min at 4°C, and the resulting pellets were washed with ice cold ethanol 70%-DEPC, centrifuged again at 13,000 rpm for 10 min at 4°C and air dried. Finally, the pellets were dissolved in 50–200 μl H_2_O-DEPC and stored at -20°C until use.

RNA concentrations and its purity were estimated from the A260/280 absorbance ratio with a NanoDrop (Thermo Scientific, Wilmington, DE, USA), considering the ideal absorbance ratio (1.8 ≤ A260/280 ≤ 2.0) and 1% agarose gel was run to visualize the integrity of the RNA.

### cDNA Synthesis

Approximately 5 μg of RNA were treated with DNase using the TURBO DNAfree^TM^ Kit (Applied Biosystems, Foster City, CA, USA), according to the manufacturer instructions. cDNA was synthesized using High-Capacity cDNA Reverse Transcription kit (Applied-Biosystems, Foster City, CA, USA) according to the manufacture’s manual.

### qPCR Conditions and Analysis

qPCR reactions were performed with 7300 System (Applied Biosystems, Foster City, CA, USA) using SYBR^®^ Green. Each reaction was performed in 20 μl containing 10 μl of 2 X Power SYBR Green PCR Master Mix (Life Technologies), 0.2–0.3 μM primers and cDNA samples diluted 1:20. Each qPCR reaction was performed in triplicate. Reactions were run using the cycling parameter described previously (Reid et al., 2006) and the qPCR data were analyzed by the 2^-ΔΔCt^ method (Pfaﬄ, 2001). In order to analyze the qPCR data, *Act11* gene was used as housekeeping to determine the relative expression level of the other genes analyzed in this work (Borges et al., 2012). *T. velutinum* T028 strain was selected as reference strain in this study based on its positive effects on bean phenotype with and without *R. solani* infection (data no published). For the determination of qPCR efficiency of each primer pairs, a standard curve was performed using the following cDNA dilutions: 1:4, 1:16, 1:64; 1:256 and 1:1024. Every measurement was made in triplicate. The corresponding qPCR efficiencies (E) were calculated for every primer pair with the software 7300 System SDS software (Applied Biosystems, Foster City, CA, USA) according to the equation E = (10^-1/slope^ - 1) × 100 (Rutledge and Stewart, 2008).

The significance of the differences between the gene expressions levels were compared by the Student’s *t*-test using SAS (SAS Institute Inc., 2004, Cary, NC, USA).

## Results

### Selection of Putative Bean Defense-Related Genes

**FIGURE 2:**
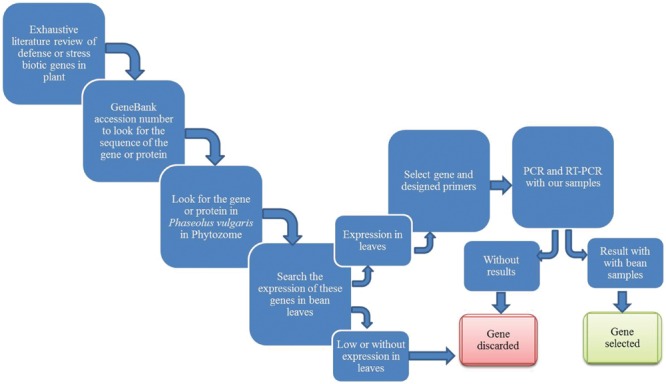
**Schematic representation of the work flow set up in the present work to select bean genes involved in plant defense**.

Following an exhaustive and systematic analysis, summarized in the **Figure 2**, several bean genes were selected for their expression analysis in leaves from bean plants grown in interaction with *T. velutinum* and infected or not with *Rhizoctonia solani*. Thus, as result of the search in the literature, 48 genes were firstly found, showing stress and/or defense response (**Table 1**). Only those genes that resulted to be expressed in *P. vulgaris* leaves, based on transcriptomic data reported in the Phytozome database^1^, were considered for qPCR expression analysis in leaves. The genes for which we confirmed expression in leaves were considered for further analyses.

**Table 1:** Genes selected for stress and/or defense response and their empirical expression in *Phaseolus vulgaris* leaves.

Id	Gene	Accession number	Functional annotation	NCBI Phytozome
**Pereira et al., 2014**				
1	*Chit*	AY357300.2	Chitanase	
2	*Glu1*	DQ093563.1	β-1,3-glucanase	
3	*Pod3*	AF485265.1	Peroxidase	
4	*PR3*	TC18606	Chitinase class I	Phvul.009G116600
5	*Lox1*	U76687.2	Lipoxygenase	
**Upchurch and Ramirez, 2010**				
6	*PPO*	EF158428	Polyphenol oxidase	
7	*PR10*	AJ289155	Stress-induced ribonuclease-like protein	
8	*PR12*	BU964598	Defensin precursor	
9	*MMP2*	AY057902	Matrix metalloproteinase 2	
10	*CHS*	X53958	Chalcone synthase	
11	*AOS*	DQ288260	Allene oxide synthase	
12	*HPL*	AW733791	Hydroperoxide lyase	Phvul.005G116800
13	*LOX2*	D13949	Lipoxygenase 2	Phvul.005G156700
14	*LOX7*	U36191	Lipoxygenase 2	Phvul.005G156900
15	*IPER*	AF007211	Basic peroxidase	Phvul.009G215000
**Borges et al., 2012**				
16	*PR16a*	CB540239	Germin-like protein 8	Phvul.010G129900
17	*PGIa*	CB542106	Polygalacturonase-inhibitor-like protein	
18	*MAPKK*	CB543156	MEK map kinase kinase	
19	*PROF*	CB543496	Profilin	
20	*CNGC2*	CB542582	Cyclin nucleotide-gated ion channel 2	Phvul.008G036200
**Guerrero-González et al., 2011**				
21	*PR1*	HO864272	Pathogenesis related protein 1	Phvul.003G109100
22	*PR2*	HO864270	Pathogenesis related protein 2	Phvul.003G109200
23	*PR4*	HO864354	Pathogenesis related protein 4	Phvul.006G102300
24	*PR10*	HO864271	Pathogenesis related protein 10)	
25	*LTP2*	HO864366	Lipid-transfer protein 2	
26	*SIP*	HO864290	Syringolide-induced protein B13-1-9	
27	*DAAP*	HO864358	Defense associated acid phosphatase	
28	*CHI*	HO864289	Chalcone isomerase	
29	*hGS*	HO864377	Homoglutathione synthetase	Phvul.006G094500
30	*aDO1*	HO864351	Alpha- dioxygenase 1	
31	*CPRD14*	HO864341	CPRD14 protein	
32	*OPR5*	HO864304	12-oxophytodienoic acid 10, 11-reductase	
33	*GST22*	HO864275	Glutathione S-transferase 22	
34	*CPRD8*	HO864396	CPRD8 protein	
35	*UDPGT*	HO864301	UDP-glucosyl transferase 72E1	
36	*ERD15*	HO864375	ERD15 protein	
37	*GTSa*	HO864392	2,4-D inducible glutathione S-transferase	Phvul.002G241400
38	*GST15*	HO864369	Glutathione S-transferase 15	
**Gallou et al., 2009**				
39	*GST1*	J03679	Gluthatione-S-transferase 1	
**Lehtonen et al., 2008**				
40	*TSI-1*	BQ121547	TSI-1 protein	
41	*Lip*	BQ112158	Lipase-like protein	
42	*Amintransf2*	BQ517030	Aminotransferase 2	Phvul.006G029100
**Bakshi and Oelmüller, 2014**				
43	*WRKY33*	NM129404.3	WRKY transcription factors	Phvul.008G090300
**Vellicce et al., 2006**				
44	*CH5b*	FE897014.1	Endochitinase precursor	Phvul.009G116500
**Lorenzo et al., 2003**				
45	*ERF1*	AF076277	Ethylene-Responsive Transcription Factor 1	Phvul.007G127800
**Moffat et al., 2012**				
46	*ERF5*	At5g47230	Ethylene-Responsive Transcription Factor 5	Phvul.002G055700
**Kim and Hwang, 2014**				
47	*PAL1*	KF279696	Phenylalanine and histidine ammonia-lyase	Phvul.001G177800
**Sharma et al., 2013**				
48	*OSM34*	At4g11650	Osmotin-like protein	Phvul.002G155500

As result, from the 48 genes selected for their involvement in bean stress and/or defense responses, only 19 were selected which showed a detectable level of expression in bean leaves.

The selected genes can be included in nine different groups (**Table 2**): (i) involved in the regulation of the balance between necrotrophic and biotrophic pathogen responses: *WRKY33* (WRKY transcription factor) (NM129404.3) (Bakshi and Oelmüller, 2014); (ii) pathogenesis related genes: *PR1* (pathogenesis related 1) (HO864272) (Guerrero-González et al., 2011), *PR2* (β 1-3 endoglucanase) (HO864270) *(*Guerrero-González et al., 2011), *PR3* (chitinase class I) (TC18606) (Pereira et al., 2014), *PR4* (pathogenesis related 4) (HO864354) (Guerrero-González et al., 2011), *PR16a* (germin.like protein 8) (CB540239) (Borges et al., 2012), *IPER* (basic peroxidase) (AF007211) (Upchurch and Ramirez, 2010), *PPO* (polyphenol oxidase) (EF158428) (Upchurch and Ramirez, 2010); (iii) related with the ethylene signaling pathway: *ERF1* (ethylene-responsive transcription factor 1) (AF076277) (Lorenzo et al., 2003), *ERF5* (ethylene-responsive transcription factor 5) (At5g47230) (Moffat et al., 2012), and *CH5b* (endochitinase precursor) (FE897014.1) (Vellicce et al., 2006); (iv) involved in phytoalexin biosynthesis: *PAL1* (phenylalanine and histidine ammonia-lyase) (KF279696) (Kim and Hwang, 2014); (v) related in osmotin biosynthesis: *OSM34* (osmitin-like protein) (At4g11650) (Sharma et al., 2013); (vi) involved in Ca^2+^ signaling: *CNGC2* (cyclic nucleotide-gated ion channel 2) (CB542582) (Borges et al., 2012); (vii) needed for antimicrobials and oxylipins (defense signaling molecules): *HPL* (hydroperoxide lyase) (AW733791) (Upchurch and Ramirez, 2010), *Lox2* (lipoxygenase 2) (D13949) (Upchurch and Ramirez, 2010), *Lox7* (lipoxygenase 2) (Upchurch and Ramirez, 2010); (viii) *GSTa* (2,4-D inducible glutathione *S*-transferase) (HO864392) (Guerrero-González et al., 2011); and (ix) *hGS* (homoglutathione synthetase) (HO864377) both related with oxidative stress (Guerrero-González et al., 2011).

**Table 2:** Common bean sequences used for primer design for RT-PCR analysis.

Gene	Functional annotation	NCBI Phytozome	Forward/Reverse	Efficiency Reference
**Reference genes**				
*Act11*	Actin-11	Phvul.008G011000	TGCATACGTTGGTGATGAGG	1.084
			AGCCTTGGGGTTAAGAGGAG	
*Ukn1*	Unknown	Phvul.011G023200	ATTCCCATCATGCAGCAAAG	0.937
			AGATCCCTCCAGGTCAATCC	
**Balance between necrotrophic and biotrophic pathogen responses**				
*WRKY33*	WRKY transcription factors	Phvul.008G090300	TTTCACAGGACAGGTTCCAGC	0.938
			CCTTTGACAGAAATGACTGAAGGA	
**Pathogenesis related genes**				
*PR1*	Pathogenesis Related 1	Phvul.003G109100	TGGTCCTAACGGAGGATCAC	1.094
			TGGCTTTTCCAGCTTTGAGT	Mayo et al., 2015
*PR2*	Beta 1-3 Endoglucanase	Phvul.003G109200	GTGAAGGACGCCGATAACAT	1.048
			ACTGAGTTTGGGGTCGATTG	Mayo et al., 2015
*PR3*	Chitinase class I	Phvul.009G116600	TGGAGTTGGTTATGGCAACAA	1.034
			ATTCTGATGGGATGGCAGTGT	
*PR4*	Pathogenesis-related 4	Phvul.006G102300	CGCAGTGAGTGCATATTGCT	0.922
			TGTTTGTCACCCTCAAGCAC	Mayo et al., 2015
*PR16a*	Germin-like protein 8	Phvul.010G129900	GGCAGTCTCATGGTTATGGTTT	–
			GCATGCTCAAGTCTCAACACAT	
*IPER*	Peroxidase precursor	Phvul.009G215000	GGCAAGCATTATATGGTTGAAA	–
			GATGGCAACATCCATCACTTTA	
*PPO*	Polyphenol oxidase	Phvul.008G073200	GAAGACGATGATTTGCTGGTTA	–
			AAGAAACATTTTCCTTTGTGAAA	
**Ethylene signaling pathway**				
*ERF1*	Ethylene-Responsive Transcription Factor 1	Phvul.007G127800	CGCTCTCAAGAGGAAACACTCC	0.937
			TGAATCAGAAGGAGGAGGGAAT	
*ERF5*	Ethylene-Responsive Transcription Factor 5	Phvul.002G055700	GGCTCCAAGTGGATTGAGAAC	0.932
			TCAGAATCAGATAACTACAAAGCACAA	
*CH5b*	Endochitinase precursor	Phvul.009G116500	CAGCCAAAGGCTTCTACACC	0.883
			TTGTTTCGTGAGACGTTTGC	Mayo et al., 2015
**Phytoalexins biosynthesis**				
*PAL1*	Phenylalanine and histidine ammonia-lyase	Phvul.001G177800	TGAGAGAGGAGTTGGGCACT	1.034
			TTCCACTCTCCAAGGCATTC	
**Osmotin biosynthesis**				
*OSM34*	Osmotin-like protein	Phvul.002G155500	GAACGGAGGGTGTCACAAAATC	0.927
			CGTAGTGGGTCCACAAGTTCCT	
**Involved in Ca^2+^ signaling**				
*CNGC2*	Cyclic nucleotide-gated ion channel 2	Phvul.008G036200	ATTCAATTTGCTTGGAGACGTT	0.98
			ACAGTTTTATTGAAGGCCAGGA	
**Antimicrobials and oxylipins (defense signaling molecules)**				
*HPL*	Hydroperoxide lyase	Phvul.005G116800	TCAAGGCTACATTTGTATTTCCA	0.984
			TGGTGCACATTTCTTAGTAGCAA	
*Lox2*	Lipoxygenase 2	Phvul.005G156700	ATGCAAGGCTAAAGAGATCCAA	–
			ATGGTGACAGGAGCTAAACACA	
*Lox7*	Lipoxygenase 2	Phvul.005G156900	GAAGGCTTGACTTTCAGAGGAA	–
			AACACACGAGAAGATTCAACCA	
**Oxidative stress**				
*GSTa*	2.4-D inducible glutathione S-transferase	Phvul.002G241400	AGGGAGTCACACTGGCTATGTT	1.013
			ATGTGCCATTTGCATTTTAGTG	
*hGS*	Homoglutathione synthetase	Phvul.006G094500	GTGGCTATATGGTGCGTACAAA	1.023
			GAAACAAGAATGCATCTCCTCA	
Amintransf2	Aminotransferase 2	Phvul.006G029100	TTCTTCCTTTTCTGCTCTTTCAA	–
			AGATGACAAGATGCAATGATTTTT	

*(–) Genes that empirically showing expression but showing negative qPCR results.*

However, only 14 genes were selected to the study of the expression genes because *PR16a, IPER, PPO, Lox2*, and *Lox7*, showing negative qPCR results, were finally discarded.

### Selection of a *Trichoderma* Strain to Validate the Gene Selection Strategy

**FIGURE 3:**
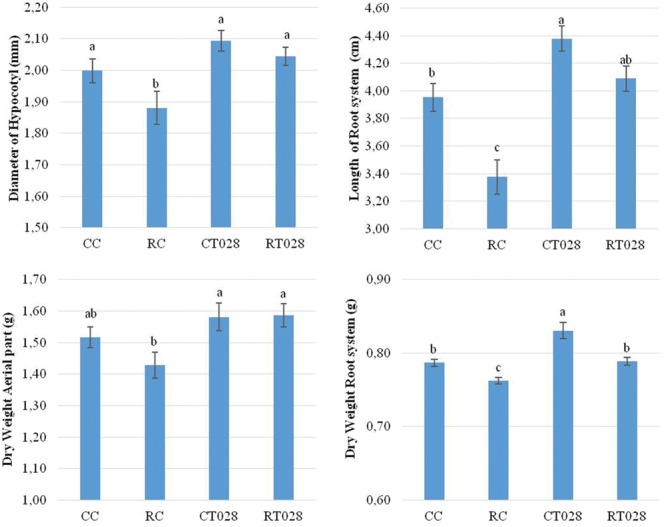
**Evaluation of the diameter of the hypocotyl **(above left)**, length of root system **(above right)** dry weight of the aerial part **(below left)** and root system **(below right)** of bean plants grown during 45 days after sowing.** [*Trichoderma velutinum* T028 without pathogen (CT028), *T. velutinum* T028 with *Rhizoctonia solani* (RT028), *R. solani* control (RC) and control without fungus (CC)]. Differences statistically significant respect to control plants (*p* < 0.05) are indicated with different letters.

*Trichoderma velutinum* T028 was the selected isolate, based on its positive effect on bean growth. Thus, plants inoculated with this strain showed a significant increase in dry weight of both aerial parts and root system, including when *R. solani* was present in the substrate (**Figure 3**). Thus, when bean plants were treated with *T. velutinum* T028 they increased respect to control plants (CC) 4.75% their diameter of hypocotyl, 10.75% their length of root system, 4.27 and 5.51% in dry weight of aerial parts and root system, respectively. When plants were infected with *R. solani*, the action of *T. velutinum* T028 caused an increased respect to the control plant with the pathogen (RC) of the diameter of hypocotyl in 8.76, 21.15% in the length of root system, and 11.05 and 3.43% in dry weight of aerial parts and root system respectively.

Based on these results, this isolate was used for further studies. In addition, this is the first report in which the effects of this strain on bean phenotype and plant gene regulation are studied.

### Effect of *R. solani* Infection on Expression of the Selected Genes. Validation of the Procedure Used to Select Bean Genes Involved in Defense Responses (Strategy Validation I)

**FIGURE 4:**
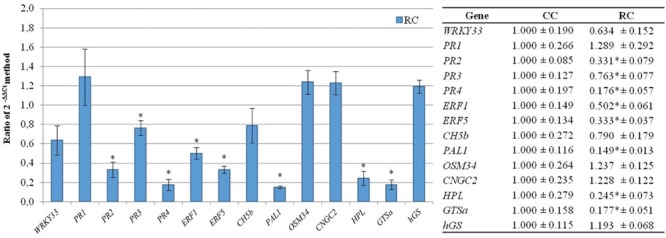
**Analysis of relative expression levels of the bean defense genes selected in the present work in bean plants infected with *R. solani versus* their levels of expression in control plants.** The data were analyzed by the 2^-ΔΔCt^ method. The differences statistically significant respect to control plants (*p* < 0.05) are indicated with an asterisk.

A significant down-regulation of expression of *PR2, PR3, PR4, ERF1, ERF5, PAL1, HPL*, and *GTSa* genes with ratios of expression ranging from 0.149 fold for *PAL1* and 0.763 fold for *PR3* was observed in bean plants grown in the presence of *R. solani* (RC) compared to control plants (CC). Conversely, expression of *PR1, OSM34, CNGC2*, and *hGS* genes was up-regulated, but with non-statistically significant differences with a ratios between 1.289 and 1.193 for *PR1* and *hGS*, respectively (**Figure 4**).

### Effect of *Trichoderma* on Expression of the Selected Genes (Strategy Validation II)

**FIGURE 5:**
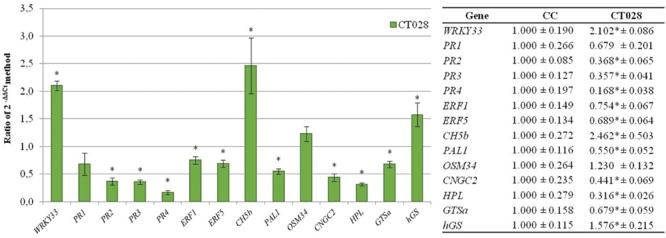
**Analysis of relative expression levels of the bean defense genes selected in the present work in bean plants treated with *T. velutinum versus* their levels of expression in control plants.** The data were analyzed as indicated in the legend to the **Figure 4**.

*Trichoderma* treatment also down-regulates expression of most of the bean defense-related genes, but at a lower level than *R. solani*. Thus, when *T. velutinum* T028 was in the substrate (CT028), *PR2, PR3, PR4, ERF1, ERF5, PAL1, CNGC2, HPL*, and *GSTa* were significantly down-regulated with expression ratios ranging from 0.168 for *PR4* to 0.754 for *ERF1*. However, *WRKY 33, CH5b*, and *hGS* were up-regulated when compared with the levels of expression in control plants, with relative expression levels between 2.462 for *CH5b* and 1.576 for *hGS* (**Figure 5**). *OSM34* was slightly but not significantly up-regulated.

### Effect of Interaction of *T. velutinum* and *R. solani* on Expression of the Selected Genes (Strategy Validation III)

**FIGURE 6:**
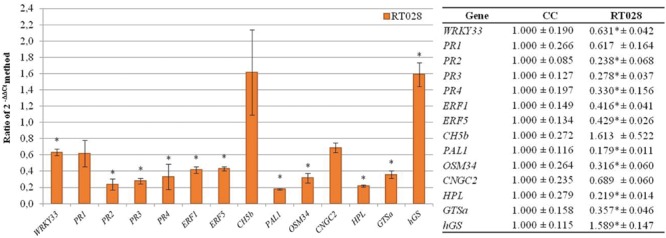
**Analysis of relative expression levels of the bean defense genes selected in the present work in bean plants infected with *R. solani* and treated with *T. velutinum versus* their levels of expression in control plants.** The data were analyzed as indicated in the legend to the **Figure 4**.

When *T. velutinum* T028 and *R. solani* (RT028) were in the substrate, the genes *WRKY33, PR2, PR3, PR4, ERF1, ERF5, PAL1, OSM34, HPL and GSTa* were significantly down-regulated with values between 0.179 for *PAL1* and 0.631 for *WRKY33.* In the case of *PR1* and *CNGC2*, they were also down-regulated but not significantly respect to control plant (C). Conversely, *hGS* was up-regulated with a significant ratio of 1.589 respect to control plants, while *CH5b* was not significantly up-regulated with a ratio of 1.613 (**Figure 6**).

## Discussion

Plants have developed some defensive strategies to perceive pathogen attack and to translate this perception into an appropriate adaptive response. During attack, plants are able to enhance their resistance (induced, acquired, hypersensitive) (Lodha and Basak, 2012). Contact with pathogenic and non-pathogenic microorganisms triggers two mechanisms: (i) SAR that is usually triggered by local infections, it provides long-term systemic resistance to pathogen attack and requires the involvement of the signal molecule salicylic acid (Durrant and Dong, 2004), and (ii) ISR that is known to result from colonization of roots by certain non-pathogenic microorganisms and is dependent on components of the jasmonic acid and ethylene signaling pathways (Shoresh et al., 2010). Then, the combination of both types of induced resistance response can protect the plant against pathogens and can even result in additive level of induced protection against pathogens through both the jasmonic acid/ethylene and salicylic acid pathways (Verhagen et al., 2006).

In the present work we developed a strategy to select genes involved in bean defense response, which would belong to those pathways, but also genes that can contribute to plant defense by other mechanisms. In this sense several previous works have described genes involved in bean defense response (Guerrero-González et al., 2011; Mayo et al., 2015). However, in the present work, by a systematic approach, 48 genes were initially considered, and 14 finally selected, which match with the criteria set up in this work: (i) they showed significant homology with previously described plant defense genes, and (ii) were expressed in bean leaves of plants treated with *Trichoderma* and/or infected with *R. solani*.

The expression of *P. vulgaris* defense-related genes was analyzed in leaves, although the interaction with *Trichoderma* and/or *R. solani* is initially produced at the root level, to determine if the signals generated in roots as result of this interaction are able to systematically stimulate the bean defense along long distance from roots to the leaves. The isolate *T. velutinum* T028 was selected following a similar strategy to that previously described (Mayo et al., 2015), and based on its positive effect on bean growth. In this work, to select a *Trichoderma* isolate, the results of the *in vitro* membrane assays and direct confrontation assays against *R. solani* were analyzed. Isolate *Trichoderma* T019 was then selected, showing a percentage of inhibition higher than 40% in the membrane assays, and/or 20% in the direct confrontation assays. This isolate also showed the best positive effects on plant phenotype among all the analyzed isolates.

WRKY transcription factors have been involved in the regulation of plant defense gene expression (Rushton and Somssich, 1998; Singh et al., 2002). Thus, WRKY33 has a role in biotic stress defense, where it regulates the balance between necrotrophic and biotrophic pathogen responses (Lippok et al., 2007; Pandey and Somssich, 2009; Birkenbihl et al., 2012). Previous studies have pointed out the involvement of *Arabidopsis* WRKY transcription factors in regulating the expression of *PR* genes by direct binding (Chen et al., 2002; Kim et al., 2006). A rapid pathogen-induced *WKRY33* expression did not require salicylic acid signaling but a downregulation of this gene involved a direct activation of jasmonic acid (Bakshi and Oelmüller, 2014). In the present case, when bean plants were in contact *T. velutinum* T028 without pathogen, the *WRKY33* gene expression was significantly up-regulated while the *PR* genes expression (*PR2, PR3* and *PR4*) was significantly down-regulated compared to expression levels in plants without *Trichoderma* treatment. In the present work, when *R. solani* was added to the substrate, expression of *WRKY33* was significantly down-regulated in plants with *Trichoderma* inoculation, while *PR2, PR3* and *PR4* were down-regulated. In the study by Mayo et al. (2015), the expression of *PR1, PR2, PR3*, and *PR4* was down-regulated when beans were inoculated with *R. solani*.

*WRKY33* is also involved in the regulation of the expression of genes modulated by components of the ethylene signaling pathway. In this work, expression of the *ERF1* and *ERF5* reached similar significant values either with or without *Trichoderma* and or *R. solani* in the substrate. This result contrasts with previous reports showing that *ERF5* was up-regulated and *WRKY33* was down-regulated in *Arabidopsis* infected with *Alternaria brassicicola* (Son et al., 2012). *WRKY33* would act as a represor of *ERF1* and *ERF5* expression. Thus, when the expression of *WRKY33* is increased, expression of *ERF1* and *ERF5* is down-regulated.

*CH5b* encodes an endochitinase precursor and it is also related with the ethylene signaling pathway. In previous works, it has been shown that, when this gene was over-expressed the *R. solani* symptoms were reduced in crops like *Nicotiana tabacum* and *Brassica napus* (Broglie et al., 1991). However, in this study, when bean plants were in contact with *R. solani*, the expression of this gene was down-regulated but not significantly, while treatment of these infected plants with *T. velutinum* resulted in its significant up-regulation. These results are in agreement with previous data, showing that the pathogen represses its expression, and the presence of *Trichoderma* induced it (Mayo et al., 2015).

*PAL* plays an important role in plant defense; it is involved in the biosynthesis of salicylic acid, which is related to plant systemic resistance (Nugroho et al., 2002; Chaman et al., 2003). *PAL* gene expression is also regulated in response to pathogen infection. In this work, the presence of *T. velutinum* and *R. solani* in the soil resulted in a significant down-regulation of this gene compared with control plants.

Osmotins have plant protective effects against pathogen infection (Narasimhan et al., 2009). In this study, when *T. velutinum* or *R. solani* were present in the soil, the expression of *OSM34* was not significantly up-regulated respect to control plants, but when both fungi were in the soil at the same time, *OSM34* was slightly but significantly down-regulated.

The *CNGC* genes can be related to early plant defense responses due to changes in ion flux, including H^+^ and Ca^2+^ influx and K^+^ and Cl^-^ eﬄux (Atkinson et al., 1996). The up-regulation of *CNGC2* can confirm the importance of ion channels for the plant resistance response (Borges et al., 2012). In this work, this gene was up-regulated when *R. solani* was present in the soil not significant. Conversely, *CNGC2* was down-regulated in plants treated with *T. velutinum*. Then, the pathogen would induce an activation of hypersensitive defense mechanisms.

Hydroperoxide lyase (*HPL*) is involved in the production of antimicrobial and defense signaling oxylipins (Noordermeer et al., 2001; Huang et al., 2010). In this study, the presence of *T. velutinum* and *R. solani*, resulted in a down-regulation of this gene expression respect to control plants. In previous works, when tomato plants were in contact with *Botrytis cinerea, HPL* expression increased 24 h after gray mold infection, but after that time the expression of this gene decreased gradually (Wan et al., 2013). In the present case, after 45 days in contact with the fungus *T. velutinum* and/or *R. solani*, its expression was down-regulated, indicating that the plant identifies *Trichoderma* and *Rhizoctonia* as two invader organisms, and some of the mechanisms activated against the presence of both are similar, independently of the final response specifically activated in the plant by each one.

*GSTa* (2,4-D inducible glutathione *S*-transferase) expression also responds to pathogen attack (Mauch and Dudler, 1993) and can be induced by molecules such as salicylic acid, methyl jasmonate, abscisic acid and H_2_O_2_ (Dixon et al., 2002; Moons, 2005). In *Gossypium arboretum*, GST provides resistance to fungal pathogens and oxidative stress (Barthelson et al., 2010). *GST* expression was up-regulated during fungal infection in barley, *Arabidopsis*, and cotton (Dowd et al., 2004; Durrant and Dong, 2004; Lu et al., 2005). However, in banana *GST* was down-regulated following *Fusarium oxysporum* f *specialis* (f. sp.) *cubense* infection (Wang et al., 2013), which is in agreement with the present case, where the expression of *GSTa* was down-regulated when *T. velutinum* and/or *R. solani* were present in the soil.

*hGS* encodes a homoglutathione synthetase that is involved in response to oxidative stress. There is not much information about the behavior of this gene in the plant. In the present study, when bean plants were in contact with *T. velutinum* and/or *R. solani*, expression of this gene was significantly up-regulated compared to control plants. In other studies, treatment of *Medicago truncatula* plants with compounds that release nitric oxide, a key signaling molecule in plants, induced expression of *GST* but not *hGS* in roots (Innocenti et al., 2007). Similarly, common bean plants treated with H_2_O_2_ showed up-regulation of *hGS* in nodules, whereas treatments with cadmium, sodium chloride, or jasmonic acid had no effect (Loscos et al., 2008).

## Conclusion

From 48 genes initially analyzed, 14 bean genes were selected in the present work and only *WRKY33, CH5b* and *hGS* showed an up-regulatory response in the presence of *T. velutinum*, the other genes were or not affected (*OSM34*) or down-regulated by the presence of this fungus. *R. solani* infection resulted in a down-regulation of most of the genes analyzed, except *PR1, OSM34* and *CNGC2* that were not affected, and the presence of both, *T. velutinum* and *R. solani*, up-regulates *hGS* and down-regulates all the other genes analyzed, except *CH5b* which was not significantly affected.

As conclusion, the strategy described in the present work has been shown to be effective to detect genes involved in plant defense, which respond to the presence of a BCA or to a pathogen and also to the presence of both. The selected genes showed significant homology with described plant defense genes and they are expressed in bean leaves of plants treated with *T. velutinum* and/or infected with *R. solani*. The proposed strategy will be very useful in studies about the interaction of bean with pathogens and biocontrol fungi.

## Author Contributions

PC and SG conceived the research. SM, OG-L, and AR-G designed the research. SM, OG-L, and AR-G conducted the experiments. SM, PC, and SG analyzed data. SM, PC, SG, EC, and FS interpreted the data. SM, PC, SG, EC, and FS wrote the manuscript. All authors were agreed to be accountable for all aspects of the work in ensuring that questions related to the accuracy or integrity of any part of the work are appropriately investigated and resolved. All authors critically revised the manuscript. All authors approved the final version to be published.

## Conflict of Interest Statement

The authors declare that the research was conducted in the absence of any commercial or financial relationships that could be construed as a potential conflict of interest.

The reviewer EVG declared a past co-authorship with one of the authors SG to the handling Editor, who ensured that the process met the standards of a fair and objective review.

## Footnotes

1 http://phytozome.jgi.doe.gov/pz/portal.html
